# P-1659. Clinical and Safety Outcomes of Uniform Thrice-Weekly Daptomycin Dosing Based on Adjusted Body Weight in Patients Receiving Intermittent Hemodialysis

**DOI:** 10.1093/ofid/ofae631.1825

**Published:** 2025-01-29

**Authors:** David T Adams, Satwinder S Kaur

**Affiliations:** Texas Health Harris Methodist Hospital Fort Worth, Fort Worth, Texas; Texas Health Harris Methodist Hospital Fort Worth , Dallas, Texas

## Abstract

**Background:**

Limited evidence exists on optimal dosing of daptomycin (DAP) in patients receiving intermittent dialysis (HD). Current labeling recommends every 48 hour (q48h) dosing which often causes additional DAP doses to be given throughout the week when the schedules become unaligned. Alternatively, current recommendations for thrice weekly dosing suggest a larger dose for the 72-hour interdialytic interval, which makes operationalization difficult within current inpatient electronic systems.

**Methods:**

A retrospective observational cohort study comparing clinical and safety outcomes of adult patients receiving DAP with HD was performed between 01/2021 and 04/2024. Updated dosing recommendations for DAP with HD was modified on 1/2023 to indication based uniform adjusted body weight (AdjBW) dosing with each HD session compared to the previous recommendation of q48h based on total body weight. Clinical outcomes included microbiologic recurrence and escalation of dose or antibiotic therapy. Safety outcomes included CK elevation and development of rhabdomyolysis. Patients were excluded if they were on DAP prior to admission or received < 2 DAP doses.

**Results:**

In total, 79 patients met inclusion. BMI, acuity of infection, length of stay, and duration of therapy were similar between the q48h (n=42) and the with each HD groups (n=37). There was a significant reduction in total weekly AdjBW DAP received in the with each HD group (23.8mg/kg vs 28.8 mg/kg, p < .0001) despite a higher utilization of the 8 mg/kg strategy (64.9% vs 26.2%). There was no significant difference in the clinical failure (5.4% vs 4.8%, *p* = 0.90) or safety outcomes (0% vs 6.5%, *p* = 0.12) between the post- and pre-groups, including after propensity matching based on BMI, AdjBW dose received, and receipt of source control.

**Conclusion:**

Utilizing uniform DAP doses with each HD compared to standard q48h frequency in patients receiving HD did not result in worse clinical or safety outcomes while reducing the overall weekly exposure to DAP. This strategy further supports the use of AdjBW as the preferred dosing weight for DAP, including in HD patients, who have been underrepresented in previous studies. This method could also be useful in the outpatient setting to help optimize DAP exposure.

**Disclosures:**

**All Authors**: No reported disclosures

